# The policy consequences of defining rewilding

**DOI:** 10.1007/s13280-021-01560-8

**Published:** 2021-05-13

**Authors:** Henrike Schulte to Bühne, Nathalie Pettorelli, Michael Hoffmann

**Affiliations:** 1 Institute of Zoology, Zoological Society of London, Regent’s Park, London, NW1 4RY UK; 2 Science and Solutions for a Changing Planet DTP and the Department of Life Sciences, Imperial College London, Buckhurst Road, Ascot, SL5 7PY UK; 3 Conservation and Policy, Zoological Society of London, Regent’s Park, London, NW1 4RY UK

**Keywords:** Human-nature relationships, Rewilding, Wilderness, Wildness

## Abstract

More than 30 years after it was first proposed as a biodiversity conservation strategy, rewilding remains a controversial concept. There is currently little agreement about what the goals of rewilding are, and how these are best achieved, limiting the utility of rewilding in mainstream conservation. Achieving consensus about rewilding requires agreeing about what “wild” means, but many different definitions exist, reflecting the diversity of values in conservation. There are three key debates that must be addressed to find a consensual definition of “wild”: (1) to which extent can people and “wild” nature co-exist?; (2) how much space does “wild” nature need? and (3) what kinds of “wild” nature do we value? Depending on the kinds of “wild” nature rewilding aims to create, rewilding policy will be faced with managing different opportunities and risks for biodiversity and people.

**Supplementary Information:**

The online version contains supplementary material available at 10.1007/s13280-021-01560-8.

## Introduction

Rewilding has emerged as a captivating, but controversial, concept in conservation (Pettorelli et al. 2019). It is currently used as an umbrella term for a wide range of conservation activities, from accepting natural vegetation succession on abandoned agricultural land to translocating functional analogues of extinct species to restore trophic networks (see Supplementary Materials). There has been extensive debate in the scientific literature about what rewilding is, what its goals are, and how they can best be achieved, across a large range of natural and social sciences (e.g. in conservation and restoration science: Pettorelli et al. 2018, Hayward et al. 2019; environmental philosophy and history: Jørgensen 2015, Prior and Ward 2016; forestry science: Dandy and Wynne-Jones 2019). Depending on how rewilding is defined, it aims to increase “wildness” of nature, regenerate ecosystem function (sensu Pettorelli et al. 2018), develop self-sustaining ecosystems, or achieve a combination of these. Environmental activists have also engaged with the term, proposing different, and often controversial, visions based on rewilding (e.g. Foreman 2004; Monbiot 2013). In addition, rewilding has attracted significant attention by stakeholders such as land use policy makers and landowners (e.g. Wentworth and Alison 2016), not all of which has been positive (Jones and Comfort 2019; BBC 2019).

The amount of debate rewilding attracts is not surprising, as its perceived risks and opportunities are significant. Proponents of rewilding tend to highlight the shortcomings of common biodiversity conservation strategies, arguing that rewilding represents a bold, proactive approach needed to safeguard biodiversity in the 21st century. They describe rewilding as an effective way to address the biodiversity crisis in an age of widespread anthropogenic global change by increasing the intrinsic resilience and transformative capacity of nature (e.g. Perino et al. 2019; du Toit and Pettorelli 2019). Rewilding is also presented as a positive new way to frame conservation for people, away from narratives of “managing declines” towards more hopeful accounts of a nature that can recover by itself (e.g. Schepers and Jepson 2016; Torres et al. 2018). However, rewilding has also large perceived risks (Maller et al. 2019; Perino et al. 2019). Some versions of rewilding focus on creating large spaces with minimal human interference and artefacts (e.g. Soulé and Noss 1998), and so could be used to legitimise the exclusion of people, their history, and their current economic and cultural activities (Jørgensen 2015; Deary and Warren 2019; Pieck 2019). Many versions of rewilding emphasise the recovery of trophic networks via species translocations. Where this includes predators, it could threaten human safety or that of their livestock and exacerbate human-wildlife conflict (O’Rourke 2019). Negative knock-on effects on extant biodiversity and ecosystem functioning can also occur from translocating other types of species, including plants (Cao et al. 2010, Delibes-Mateos et al. 2019). Passive rewilding, i.e. letting natural processes re-establish by withdrawing human activities, could also have unintended negative consequences. These include the loss of open habitat specialists due to passive regeneration of forests (e.g. Herrando et al. 2016), or risking soil loss if wild vegetation cannot establish on abandoned fields (Khanal and Watanabe 2006).

In parallel to these discussions about the opportunities and risks of rewilding, rewilding has catalysed a significant conservation movement, especially in the Global North, with many organizations now practicing and promoting rewilding as part of their conservation strategies. Mirroring the diversity of definitions in the scientific literature, these organisations take different approaches to rewilding. In North America, the focus is often on protecting carnivores in large, connected landscapes (Wildlands Network 2020), whereas European approaches focus on passive rewilding on abandoned farmland and on reintroducing large herbivores (Jones and Comfort 2019). In Australia, rewilding projects emphasise the restoration of native predator populations and small mammal communities by controlling invasive predators (Sweeney et al. 2019). Several conservation NGOs have also started to implement rewilding projects (e.g. Wildlife Conservation Society’s Rewilding the Rockies project, WCS 2020; Summit to Sea project in Wales, County Times 2020) and to develop policies or institutional strategies anchored on the tenets of rewilding (e.g. Woodland Trust 2017; Durrell Wildlife Conservation Trust 2020). Other conservation NGOs have started promoting rewilding as a principle in conservation legislation (European Habitats Forum 2020), and intergovernmental entities such as the Convention on Biological Diversity (CBD) have been exploring the potential for rewilding to contribute to biodiversity conservation goals (CBD 2014), though at the time of writing there was no explicit reference to rewilding in the zero draft of the post-2020 Global Biodiversity Framework (CBD 2020). Clearly, the concept of rewilding has entered the vocabulary of mainstream conservation. However, relatively little legislation, policy and best-practice guidance for rewilding exists to date, and indeed there is still limited explicit reference to rewilding in much international and national legislation (Cretois et al. 2019).

If rewilding is to be fully integrated into national and international biodiversity conservation frameworks, there needs to be a clear vision of what it is, but there is currently little agreement on the definition of rewilding among different stakeholders (sensu Wallace et al. 2016). Efforts to build such a consensus among experts and practitioners are ongoing; for example, the International Union for Conservation of Nature’s (IUCN) Commission of Ecosystem Management (CEM) tasked a dedicated group within its constituency to develop a conceptual and methodological framework for rewilding, and an IUCN-wide Rewilding working group has been approved recently. Agreeing on an operational definition of rewilding would facilitate making appropriate rewilding policy in three ways. First, clarifying what types of projects fall under the umbrella of rewilding would make it easier to develop principles and guidelines to help inform rewilding initiatives and ensure that these deal with uncertainty and risks arising from such projects. Second, it would support the development of meaningful objectives and success indicators for rewilding projects, thus ensuring rewilding projects can be managed effectively. Finally, it would enable biodiversity conservation decision makers to assess whether rewilding is an appropriate tool to achieve their goals in a given situation, i.e. aid the decision of when to choose rewilding rather than other strategies to protect and promote biodiversity.

Many of the debates around rewilding focus on the perceived benefits and drawbacks of different strategies used to rewild ecosystems, with the spotlight on the rewiring of trophic networks via the translocation (sensu IUCN/SSC 2013) of large animals. However, the merits of different rewilding strategies can only be assessed in relation to the aims they are trying to achieve, so discussing which tools should be used in rewilding is unlikely to lead to clarity about what rewilding is. Instead, how rewilding is defined will ultimately depend on what is defined as “wild”, and thus desirable, by rewilding stakeholders. Yet stakeholders currently disagree over the meaning of “wild”, which impedes finding a consensus on what rewilding is. The different meanings of “wild”, as well as related concepts such as “wildness” or “wilderness”, are socially constructed concepts (Frazier 2010), and cannot be understood without reference to the diversity of knowledge, morals and norms underpinning current thinking on conservation (Mace 2014; Hall 2014).

Existing definitions of “wild” can be classified into two broad positions: on the one hand, wild describes wilderness, i.e. large, remote, pristine areas where people have (or had) minimal impact; on the other hand, wild describes wildness, which is the autonomy of non-human actors in a system, e.g. the ability of a wild herbivore to freely choose where to go and what to forage (European Commission 2013; De Cózar-Escalante 2019; Ward 2019). Importantly, “wilderness” always refers to a place, whereas “wildness” is a condition that can apply to a range of entities, such as processes and populations (Prior and Brady 2017; De Cózar-Escalante 2019).

However, reducing discussions of the definition of “wild” to the binary of wildness versus wilderness brushes over some of the underlying debates over what qualities stakeholders value in “wild” nature that have important consequences for rewilding policy. Here, to contribute to the process of finding consensus around rewilding, we identify three key questions that need to be addressed: (1) can “wild” nature and people co-exist?; (2) how much space does “wild” nature need?; and (3) what kinds of “wild” nature do we value over others? Rather than positing a viewpoint on each of these questions, our contribution focuses on highlighting some of the policy-relevant consequences of different answers.

## What is “wild”?

### Can “wild” nature and people co-exist?

A key tension exists between those positions that define “wild” as synonymous with the absence of human impact and those that define “wild” as non-human autonomy, which can in principle co-exist with human presence to some degree (Ward 2019; Table 1). Whether “wild” is synonymous with absence of people has significant policy-relevant consequences for the role that people can play in rewilded landscapes—including the range and intensity of human activities that are acceptable. This debate does not focus on those human activities that are intended to aid the process of rewilding per se (see Supplementary Materials), but rather any consumptive and non-consumptive use of nature that could occur in a rewilded ecosystem, ranging from land use for agriculture, to hunting, to recreational activities such as tourism.

**Table 1:** A non-exhaustive list of opportunities and risks of different definitions of “wild” for rewilding policy

Dimension of “wild”	Potential position	Opportunities	Risks
Role of people	People and “wild” nature cannot, or have limited opportunity to, co-exist in a shared space	Minimal anthropogenic pressure on biodiversity Potential for entirely self-sustaining ecosystems	Exclusion of people and their artefacts from rewilded areas likely to be necessary Reduced engagement and support from local communities who could benefit from rewilding and rewilded sites
	People and “wild” nature can co-exist in a shared space	Allows integrating (some) human activities and legacies in rewilded sites More locations suitable for rewilding	Potential for increased human-wildlife conflict in rewilded sites where people are present
Spatial scale	All scales	Rewilding possible in areas with a high degree of intensive human land use (e.g. cities)	Smaller sites may not be able to deliver on a large range of ecosystem functions
	Only large sites	Able to support more species and ecosystem functions	Limited scope and utility for rewilding in densely populated countries
Acceptable “wild” nature	Historical ecosystems	Allows identifying reference systems, and hence relatively easy to define and measure success	Similar to restoration; means rewilding would have little additional value as a conservation approach
	Any ecosystem that can autonomously respond to external and internal change, including novel ecosystems	Can cope with inevitable environmental and ecological change	More difficult to define what successful rewilding looks like

Definitions of “wild” that posit that it cannot include people imply that wild ecosystems are pristine, “natural”, and self-regulating. Consequently, (re-)creating “pristine” ecosystems means reducing or removing past and present human impacts, and this conceptualisation of “wild” is often associated with removing human artefacts or settlements from an area protected because of its “wild” quality (Cronon 2003). This position values “wilderness” for its perceived benefits for biodiversity, and argues that rewilding is most impactful when it sets ambitious goals for nature (Genes et al. 2019; see also the definition of wilderness as “untrammelled by man” in the US Wilderness Act from 1964). Under this vision, even relatively low-impact human activities can modify ecosystem structure, composition, and function (e.g. Suraci et al. 2019), hampering ecosystems from achieving self-sustenance. Proponents of this form of rewilding also suggest that separating rewilding and human activities in space could reduce the exposure of people to any risks of rewilding, such as human-wildlife conflict. This could reduce the potential for conflict between communities living and using land intended for a rewilding project, who are directly exposed to the risks of rewilding, and communities living further away, who are not exposed but may still be in favour of rewilding (e.g. Bauer et al. 2009).

However, a major criticism of this vision is that it could be used to argue for the exclusion of people, their traditional cultural practices, and their livelihoods (Hayward et al. 2019), and to eradicate people’s history from landscapes (Hall 2014; Deary and Warren 2019), threatening their identities. This has created strong opposition to rewilding among some stakeholders, sometimes resulting in the abandonment of rewilding projects (Pieck 2019). Trying to (re-)create ecosystems that have no human influence has also been criticised from an ecological perspective, given that human impacts on the environment are ubiquitous and truly pristine areas cannot not exist or be maintained in many places due to climate change and other anthropogenic global change (e.g. Hobbs et al. 2006). Arguably, modern humans and their ancestors have been functional components of ecosystems for millions of years, so that complete removal of our species could have unintended negative side effects (Root-Bernstein and Ladle 2019). More recently, wild species such as Australian Brush-turkeys (Alectura lathami) in Australia and White-tailed Deer (Odocoileus virginianus) in the US (Maller et al. 2019), as well as hedgehogs in the UK (Pettett et al. 2017), have been shown to benefit from resources found in close to human settlements, such as suburban gardens, showing that “wild” nature can closely interact with human-dominated spaces. It thus seems unlikely that many stakeholders will be able to support a version of rewilding that is primarily focussed on removing human influence and all past and present anthropogenic impacts.

An alternative conceptualisation of “wild” defines it as non-human autonomy. This definition is not focussed on separating “wild” nature from humans, and is compatible with interdependency of human–environment relationships (which is often found in environmental concepts outside the Western tradition, e.g. “kincentric ecology”, Salmón 2000). Following this definition, the mere presence of people and their activities does not automatically disqualify a place or population from having “wild” aspects (Scotney 2014). Instead, nature can be “wild” in ecosystems in which humans exist and modify ecosystem structure, composition and function, as long as nature retains significant autonomy. Such “wild” ecosystems could include traditional livestock grazing systems in Europe, which have created open landscapes that support highly biodiverse communities (e.g. Niedrist et al. 2009), or grassland ecosystems in Africa, which have been co-developing with anthropogenic fire for millennia (Archibald et al. 2012). This conceptualisation of “wild” has direct consequences for rewilding policy, because it maintains the possibility of significant human activity in and with rewilded landscapes (e.g. Carter and Linnell 2016), and thus avoids some of the potential negative consequences of excluding people from rewilded systems.

The degree to which cultural and livelihood activities can occur in or near rewilded sites will greatly affect the economic, social and cultural risks, costs and benefits of rewilding (e.g. Pedersen et al. 2020). One immediate consequence of allowing more types and higher intensity of human activities in rewilded spaces and more access for people is that more areas will be potentially suitable for rewilding. It may also be easier to gain buy-in from local stakeholders if rewilding is seen as compatible with land management approaches that value cultural and economic significance of landscapes (Community Land Scotland 2017). In addition, rewilding proponents and local communities have cited potential benefits to marginalised rural communities, in economic and social terms (“enlivenment”: Vasile 2018; Jones and Comfort 2019), as key benefits of rewilding projects. It may be easier for communities to access these benefits if they are physically close to rewilded places and can use their natural resources to some degree. Limiting rewilding to remote or less accessible places could potentially reduce the benefits from rewilding especially to urban communities, at a point in time when global levels of urbanization are growing (Maller et al. 2019).

If the aim is to give nature more autonomy, then arguably not all human activities can be allowed in a rewilded site or landscape, especially where activities could significantly impact rewilding goals; at the same time, many stakeholders stress that humans need, and should not be erased from, rewilded nature. A key question is thus in which way different human activities and rewilding can be integrated to achieve rewilding goals without threatening human welfare. The answer to this question will depend on both the kind of human activity in question, and the goals for biodiversity conservation adopted by a given rewilding project.

### How much space does “wild” nature need?

Another key disagreement concerns the spatial scale at which “wild” nature can be sustained. Some argue that “wild” nature can only be sustained in large areas of land (e.g. Soulé and Noss 1998), while others argue that “wild” nature can occur in smaller spaces within a matrix of more intense human land use (e.g. Diemer et al. 2003; Table 1). The former position tends to be associated with “wilderness”, since entirely self-regulating ecosystems, where humans have no control, are likely only achievable at large scales. This argument stems from the observation that there are ecosystem components and functions that need large areas to be self-sustaining, including the persistence of species with large home ranges (but see Carter and Linnell 2016), or the development of “natural” disturbance regimes such as fires (Archibald et al. 2013). Second, due to the so-called edge effects, the impact of human activities can be experienced at some distance from the source (e.g. Reinmann and Hutyra 2017), meaning a large area is needed so that anthropogenic impacts cannot affect the dynamics of the ecosystem at its core.

This contrasts with definitions of “wild” that stress autonomy of nature, which can in principle occur in small areas alongside intensive human land uses, including even small areas in urban settings (Maller et al. 2019). Smaller areas are unlikely to support entirely self-organising ecosystems, but they could be valuable space for some autonomous processes and species (e.g. van den Bosch and Sang 2017). For instance, smaller rewilded sites could play an important ecological role as stepping stones, or temporary habitats, for some species as they travel between larger sites, for instance due to shifts in their preferred climate (Han and Keeffe 2020). Where smaller rewilding sites are integrated into landscapes with more intensive human land use, and so are more accessible, they may also provide important recreational, spiritual and educational benefits to people. For instance, ecotourism to large and remote rewilding sites may be prohibitively expensive for many people (see recent prices for ecotourism experiences, Acorn Tourism Consulting Limited 2019), whereas more local, smaller sites could provide more equitable access.

How big rewilded sites have to be thus ultimately depends on the goals that are pursued by a given rewilding project, i.e. whether the types of nature that can be achieved at a given spatial scale is considered to fall within the meaning of “wild” by the relevant stakeholders. As a result, this debate is perhaps most constructive when it asks how small-scale rewilding efforts can benefit “wild” nature and promote biodiversity conservation. It would in theory be possible to define different types or categories of rewilding sites, similar to how the IUCN protected area categories assign protected areas to one of six categories based on the primary management objective of the site (Dudley 2008). Diemer et al. (2003) refer to such a typology for urban rewilding, from “rewilding microcosms” in very small areas in, or very close to, cities such as private gardens, to larger urban rewilding areas in areas where urban infrastructure such as mining sites have been abandoned, to “urban wilderness”, areas with little or no human land use that are relatively close to urban centres. The typology highlights that rewilding site “types” can have different objectives. For instance, larger, more remote sites will be more suitable for large carnivore conservation than smaller, urban sites, whereas the latter can contribute to ecological processes and ecosystem services such as microclimate regulation and recreation. When taking a landscape approach to planning rewilding, it may be beneficial to aim for a mix of smaller and larger rewilding sites that together deliver the outcomes desired by stakeholders. A key challenge of accepting a mix of spatial rewilding scales is then determining what size and configuration of rewilding sites are required to achieve rewilding goals in different ecosystems (Thompson et al. 2018).

### What kind of “wild” nature do we value over others?

Another key debate is what kinds of “wild” ecosystem states and trajectories stakeholders prefer over others (Table 1). If wild means pristine wilderness, there is at least in theory an “ideal” ecosystem state and change trajectory, namely those it would have had if there had been no human influence at all. Definitions of “wild” that stress “pristineness” or “purity” of nature have been prevalent in Western conservation thinking since the start of the conservation movement (Cronon 1995). Following this definition, it is important to identify appropriate reference points or ecosystems (either historical or existing) to describe this “wild” state as a particular assemblage of species, ecosystem structure, and range of ecosystem functions (e.g. Hiers et al. 2012). Conservation often works to prevent or mitigate changes away from this state due to human influence. This way of describing “wild” is also in keeping with the way in which ecosystem restoration has been operating in practice (Gann et al. 2019). In a rewilding context, stakeholders who follow a definition of wild as “pristine” are likely to use historical or contemporary ecosystems as benchmarks to legitimise objectives, and value minimal human impact on the landscape (e.g. Scottish Natural Heritage 2014).

However, both within and outside restoration science, there is recognition that anthropogenic change cannot be prevented in many places (Hobbs et al. 2010). Legacies of human impact and ongoing global change, especially climate change, can make it difficult to recreate historical ecosystems at times, since they may not be able to persist in the long term (e.g. Gilman et al. 2010). Novel ecosystems may often be inevitable. Consequently, the focus has shifted to valuing the ability of “wild” nature to autonomously respond to external and internal change (Child et al. 2019, Perino et al. 2019). Under this definition, “wild” ecosystems are valued if they can adapt to external change and undergo reorganization and transition from one stable state to another (du Toit and Pettorelli 2019) without being managed by people. For instance, a wild population of animals can adapt to anthropogenic climate change through natural selection or migration. This way of thinking about “wild” ecosystems means that novel ecosystems can be wild even if they have no historical or present-day analogues, provided they develop and respond autonomously to external pressures.

Even if we value autonomous change in “wild” ecosystems, not all outcomes will be equally acceptable to all stakeholders. For instance, it is unlikely that many rewilding stakeholders, including conservationists, are going to value feral dogs (Canis lupus familiaris) for their autonomy and ecological function as predators, unlike the Grey Wolf (Canis lupus) or lynx (Lynx spp.; Vasile 2018). There will thus need to be an explicit decision by the stakeholders of a rewilding project on what ecosystem states, functions, dynamics and trajectories are acceptable. Because our understanding of long-term ecosystem dynamics is not sufficient to determine future outcomes in all cases (Norden et al. 2015), rewilding policy will have to address temporal uncertainty explicitly.

From an ecological perspective, if stakeholders value ecosystems that are able to rearrange and adapt in response to external and internal change, then desirable “wild” ecosystems or species populations are those in which ecological and evolutionary processes are effective at reorganising biota, i.e. those with high resilience and transformative capacity (e.g. Child et al. 2019). For instance, Torres et al. (2018) suggest that ecological integrity (i.e. whether an ecosystem has a natural disturbance and dispersal regime, and has high trophic complexity) shows the degree to which autonomous ecological processes are present in an ecosystem, and is thus a useful metric to assess the success of rewilding. Identifying processes that are desirable in “wild” ecosystems can be informed by looking at the past (Higgs et al. 2014), as this can for instance help inform our understanding of the factors determining biodiversity responses to climatic changes. It is unclear, however, if our understanding of long-term ecosystem dynamics is sufficient to determine such processes in all cases. Regardless of which processes are identified as contributing to ecological integrity, resilience or transformative capacity, this approach likely requires a tailored expert assessment to determine what these processes should ideally look like in a given rewilding site, and whether the rewilded ecosystem is moving towards this state. However, it is not always clear how experts determine what hypothetical ideal state a given ecosystem could achieve, i.e. which knowledge, experiences and values have come to bear on the assessment.

The risks associated with novel ecosystems may be more difficult to justify, and harder to predict, than risks arising from restoring a system to a historical precedent; it may thus be more difficult to find support from land owners and other affected stakeholders. On the other hand, it is not immediately obvious that the risks from activities that are closer to restoration, or from taking no action, are necessarily lower (Remm et al. 2019), and novel ecosystems can in fact be highly valued by stakeholders (Kaae et al. 2019). In any case, to implement rewilding ethically, objectives of a rewilding project will have to be identified in a transparent and inclusive process, with particular regard to marginalised groups. Deciding on what rewilding objectives are may expose or create conflicts between different groups of stakeholders and will likely require significant communication and mediation long before the implementation of a rewilding project.

## Conclusions

**Fig. 1:**
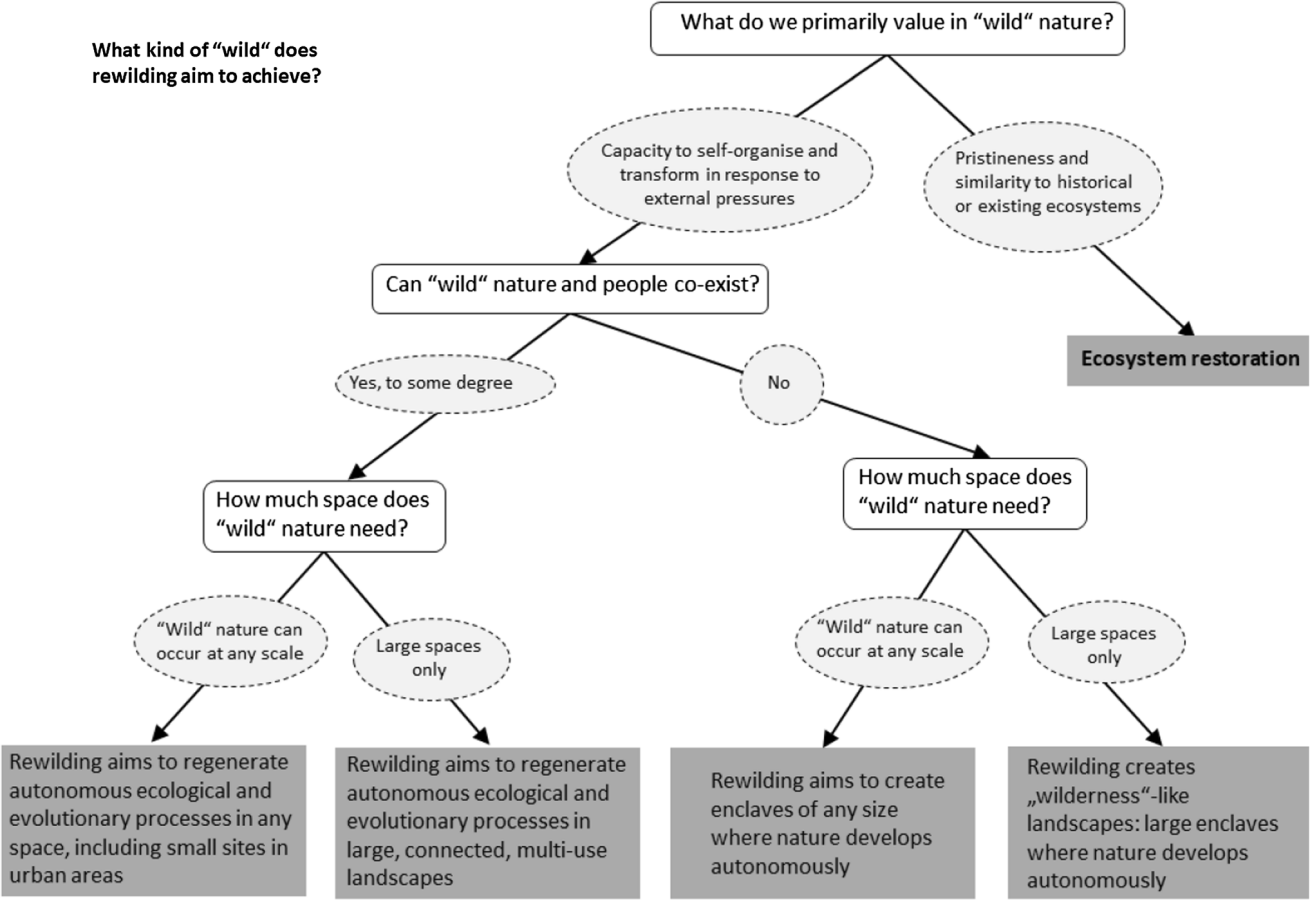
Flowchart showing the consequences of different definitions of “wild” for the concept of rewilding

What rewilding looks like in practice depends on how stakeholders define “wild” (Figure 1). This means that defining rewilding is ultimately a political and social process informed by ecological knowledge rather than determined entirely by it. The type and intensity of human activities that are possible in rewilded sites depend on the degree to which stakeholders believe “wild” nature and people can co-exist. The size and location of rewilding projects will depend largely on opinions about the space which nature needs to be truly “wild”. Finally, since “wild” nature can change along different trajectories, it is necessary to define whether we look to past or present ecosystem states, or potential for change and transformation, to define ideal “wild” nature. Consequently, each rewilding vision will create a different set of ethical, economic, and environmental challenges, and so shape the socio-ecological contexts in which rewilding is an appropriate tool for managing human–environment relationships. Addressing these challenges can only be successful, however, if there is a consensus on what rewilding is trying to achieve.

At the landscape scale, active conservation management, ecosystem restoration and rewilding could provide complementary benefits to biodiversity (e.g. van Meerbeek et al. 2019; Pedersen et al. 2020), but how rewilding will be able to complement existing tools will depend on what goals it ultimately pursues. For instance, when taking a view that humans and wild nature can co-exist in a shared space, rewilding could be a useful approach to maintaining biodiversity in places where the legacy of human actions or ongoing global change makes restoration difficult (Schläppy and Hobbs 2019). Similarly, if rewilding embraces ecosystem change and transitions to novel systems, it is unlikely to be an appropriate conservation strategy in sites that have key populations of threatened species, or remnant threatened habitats, where active conservation management is preferable. Conversely, depending on the version of rewilding that is practiced, it may be able to contribute to or facilitate achieving aims of ecosystem restoration or active conservation management, for instance by improving flows of species, nutrients or energy through the wider landscape. Achieving the best landscape-level mix of strategies would be facilitated by best-practice guidance on how to decide whether a particular ecological and socio-economic context is best suited to rewilding or other conservation strategies.

The debates surrounding rewilding have highlighted that biodiversity conservation is not ideologically homogenous. Though this has created tensions between rewilding stakeholders, it also offers a unique opportunity to discuss often implicit values and norms held by individuals and organisations involved in biodiversity conservation. If this discussion is inclusive of many different stakeholders, especially marginalised groups, rewilding could provide a productive forum for developing a forward-looking conservation paradigm fit for the 21st century.

## Supplementary Information

Below is the link to the electronic supplementary material.

Supplementary material 1 (PDF 676 kb)

## References

[CR1] Acorn Tourism Consulting Limited Lt. 2019. Entering the European market for nature and ecotourism. https://www.cbi.eu/market-information/tourism/nature-ecotourism/market-entry/#what-are-the-prices-for-nature-and-ecotourism-travel-products-on-the-european-market. Accessed 23 June 2020.

[CR2] Archibald S, Staver AC, Levin SA (2012). Evolution of human-driven fire regimes in Africa. Proceedings of the National Academy of Sciences.

[CR3] Archibald S, Lehmann CE, Gómez-Dans JL, Bradstock RA (2013). Defining pyromes and global syndromes of fire regimes. Proceedings of the National Academy of Sciences.

[CR4] Bauer N, Wallner A, Hunziker M (2009). The change of European landscapes: human-nature relationships, public attitudes towards rewilding, and the implications for landscape management in Switzerland. Journal of environmental management.

[CR5] BBC, 2019. https://www.bbc.com/news/uk-wales-49186349. Accessed 23 June 2020.

[CR6] Cao S, Tian T, Chen L, Dong X, Yu X, Wang G (2010). Damage caused to the environment by reforestation policies in arid and semi-arid areas of China. Ambio.

[CR7] Carter NH, Linnell JD (2016). Co-adaptation is key to coexisting with large carnivores. Trends in Ecology & Evolution.

[CR8] CBD (Convention on Biological Diversity), 2014. Secretariat of the Convention on Biological Diversity (2014) Global Biodiversity Outlook 4. Montréal, 155 pp.

[CR9] CBD (Convention on Biological Diversity), 2020. Zero draft of the post-2020 global biodiversity framework. https://www.cbd.int/doc/c/351d/06cd/2facb945f9d6ed93fdb22da7/wg2020-02-03-en.docx. Accessed 23 June 2020

[CR10] Child MF, Selier SJ, Radloff FG, Taylor WA, Hoffmann M, Nel L, Power RJ, Birss C (2019). A framework to measure the wildness of managed large vertebrate populations. Conservation Biology.

[CR11] Community Land Scotland. 2017. Position Paper on `rewilding’. https://www.communitylandscotland.org.uk/wp-content/uploads/2017/11/Position-Paper-on-rewilding-2017.pdf. Accessed 23 June 2020

[CR12] County Times. 2020. RSPB Cymru takes over Mid Wales Summit to the Sea project. https://www.countytimes.co.uk/news/18526360.rspb-cymru-takes-mid-wales-summit-sea-project/. Accessed 23 June 2020

[CR13] Cretois B, Linnell JD, Kaltenborn BP, Trouwborst A (2019). What form of human-wildlife coexistence is mandated by legislation? A comparative analysis of international and national instruments. Biodiversity and Conservation.

[CR14] Cronon W (2003). The riddle of the Apostle Islands. Orion.

[CR15] Dandy N, Wynne-Jones S (2019). Rewilding forestry. Forest Policy and Economics.

[CR16] Deary H, Warren CR (2019). Trajectories of rewilding: A taxonomy of wildland management. Journal of Environmental Planning and Management.

[CR17] De Cózar-Escalante JM (2019). Rewilding A Pragmatist Vindication. Ethics Policy & Environment.

[CR18] Delibes-Mateos M, Barrio IC, Barbosa AM, Martínez-Solano I, Fa JE, Ferreira CC, Pettorelli N, Durant SM, Du Toit JT (2019). Rewilding and the risk of creating new, unwanted ecological interactions. Rewilding.

[CR19] Diemer M, Held M, Hofmeister S (2003). Urban wilderness in Central Europe. International Journal of Wilderness.

[CR20] Dudley, N., Editor. 2008. Guidelines for Applying Protected Area Management Categories. Gland, Switzerland: IUCN. x + 86pp. WITH Stolton, S., P. Shadie and N. Dudley. 2013. IUCN WCPA Best Practice Guidance on Recognising Protected Areas and Assigning Management Categories and Governance Types, Best Practice Protected Area Guidelines Series No. 21, Gland, Switzerland: IUCN.

[CR21] Durrell Wildlife Conservation Trust. 2020. Rewild our world. https://www.durrell.org/wildlife/rewild-our-world. Accessed 23 June 2020

[CR22] du Toit JT, Pettorelli N (2019). The differences between rewilding and restoring an ecologically degraded landscape. Journal of Applied Ecology.

[CR23] European Commission. 2013. Guidelines on Wilderness in Natura 2000. Management of Terrestrial Wilderness and Wild Areas Within the Natura 2000 Network. Technical Report 2013-069.

[CR24] European Habitats Forum 2020. The implementation of the EU 2020 Biodiversity Strategy and recommendations for the post 2020 Biodiversity Strategy. http://d2ouvy59p0dg6k.cloudfront.net/downloads/ehf_paper_post_2020_eu_biodiversity_strategy_may2019.pdf. Accessed 23 June 2020.

[CR25] Foreman D (2004). Rewilding North America: a vision for conservation in the 21st century.

[CR26] Frazier, J.G, 2010. The call of the wild. *The Archaeology of Anthropogenic Environments*, ed. R. M. Dean. Center for Archaeological Investigations. Occasional Paper No.37.

[CR27] Gann GD, McDonald T, Walder B, Aronson J, Nelson CR, Jonson J, Hallett JG, Eisenberg C (2019). International principles and standards for the practice of ecological restoration. Second edition. Restoration Ecology.

[CR28] Genes L, Svenning JC, Pires AS, Fernandez FA (2019). Why we should let rewilding be wild and biodiverse. Biodiversity and Conservation.

[CR29] Gilman SE, Urban MC, Tewksbury J, Gilchrist GW, Holt RD (2010). A framework for community interactions under climate change. Trends in Ecology & Evolution.

[CR30] Hall, M. 2014. Extracting culture or injecting nature? Rewilding in transatlantic perspective. In *Old World and New World Perspectives in Environmental Philosophy*, 17–35. Cham: Springer

[CR31] Han, Q., and Keeffe, G. 2020. Stepping-stone city: process-oriented infrastructures to aid forest migration in a changing climate. In *Nature Driven Urbanism*, 65–80. Cham: Springer,.

[CR32] Hayward MW, Scanlon RJ, Callen A, Howell LG, Klop-Toker KL, Di Blanco Y, Balkenhol N, Bugir CK (2019). Reintroducing rewilding to restoration–rejecting the search for novelty. Biological Conservation.

[CR33] Herrando S, Brotons L, Anton M, Paramo F, Villero D, Titeux N, Quesada J, Stefanescu C (2016). Assessing impacts of land abandonment on Mediterranean biodiversity using indicators based on bird and butterfly monitoring data. Environmental Conservation.

[CR34] Hiers JK, Mitchell RJ, Barnett A, Walters JR, Mack M, Williams B, Sutter R (2012). The dynamic reference concept: measuring restoration success in a rapidly changing no-analogue future. Ecological Restoration.

[CR35] Higgs E, Falk DA, Guerrini A, Hall M, Harris J, Hobbs RJ, Jackson ST, Rhemtulla JM (2014). The changing role of history in restoration ecology. Frontiers in Ecology and the Environment.

[CR36] Hobbs RJ, Arico S, Aronson J, Baron JS, Bridgewater P, Cramer VA, Epstein PR, Ewel JJ (2006). Novel ecosystems: theoretical and management aspects of the new ecological world order. Global Ecology and Biogeography.

[CR37] Hobbs RJ, Cole DN, Yung L, Zavaleta ES, Aplet GH, Chapin FS, Landres PB, Parsons DJ (2010). Guiding concepts for park and wilderness stewardship in an era of global environmental change. Frontiers in Ecology and the Environment.

[CR38] IUCN/SSC. 2013. Guidelines for Reintroductions and Other Conservation Translocations. Version 1.0. Gland, Switzerland: IUCN Species Survival Commission, viiii + 57 pp.

[CR39] Jones, P., and Comfort, D. 2019. A commentary on rewilding in Europe. *Journal of Public Affairs*, p.e2071. 10.1002/pa.2164 PMC7300595 32837318

[CR40] Jørgensen D (2015). Rethinking rewilding. Geoforum.

[CR41] Kaae, B.C., Holm, J., Caspersen, O.H., and Gulsrud, N.M. 2019. Nature Park Amager–examining the transition from urban wasteland to a rewilded ecotourism destination. *Journal of Ecotourism*, 1–20.

[CR42] Khanal NR, Watanabe T (2006). Abandonment of agricultural land and its consequences. Mountain Research and Development.

[CR43] Mace GM (2014). Whose conservation?. Science.

[CR44] Maller C, Mumaw L, Cooke B, Pettorelli N, Durant SM, Du Toit JT (2019). Health and social benefits of living with ‘wild’nature. Rewilding.

[CR45] Monbiot, G. 2013. Feral: Searching for enchantment on the frontiers of rewilding. Penguin UK.

[CR46] Niedrist G, Tasser E, Lüth C, Dalla Via J, Tappeiner U (2009). Plant diversity declines with recent land use changes in European Alps. Plant Ecology.

[CR47] Norden N, Angarita HA, Bongers F, Martínez-Ramos M, Granzow-de la Cerda I, Van Breugel M, Lebrija-Trejos E, Meave JA (2015). Successional dynamics in Neotropical forests are as uncertain as they are predictable. Proceedings of the National Academy of Sciences.

[CR48] O’Rourke, E. 2019. The raptor and the lamb: reintroduction of carnivores in agricultural landscapes in Ireland. In *Natural Resource Conflicts and Sustainable Development*, 69–83. London: Routledge.

[CR49] Pedersen PBM, Ejrnæs R, Sandel B, Svenning JC (2020). Trophic rewilding advancement in anthropogenically impacted landscapes (TRAAIL): A framework to link conventional conservation management and rewilding. Ambio.

[CR50] Pettett CE, Moorhouse TP, Johnson PJ, Macdonald DW (2017). Factors affecting hedgehog (*Erinaceus europaeus*) attraction to rural villages in arable landscapes. European Journal of Wildlife Research.

[CR79] Perino, A., H.M. Pereira, L.M. Navarro, N. Fernández, J.M. Bullock, S. Ceauşu, A. Cortés-Avizanda, R. van Klink, et al. 2019. Rewilding complex ecosystems. *Science* 364: eaav5570. 10.1126/science.aav5570 31023897

[CR51] Pettorelli N, Schulte to Bühne H, Tulloch A, Dubois G, Macinnis Ng C, Queirós AM, Keith DA, Wegmann M (2018). Satellite remote sensing of ecosystem functions: Opportunities, challenges and way forward. Remote Sensing in Ecology and Conservation.

[CR52] Pettorelli, N., Durant, S. M., and Du Toit, J. T. 2019. Rewilding: A captivating, controversial, 21st century concept to address ecological degradation in a changing world. In *Rewilding*, eds Pettorelli, N., Durant, S. M., and Du Toit, J. T. Cambridge: Cambridge University Press.

[CR53] Pieck, S.K., 2019. Conserving novel ecosystems and layered landscapes along the inter-German border. *Landscape Research*, 1–13.

[CR54] Prior J, Ward KJ (2016). Rethinking rewilding: A response to Jørgensen. Geoforum.

[CR55] Prior J, Brady E (2017). Environmental aesthetics and rewilding. Environmental Values.

[CR56] Reinmann AB, Hutyra LR (2017). Edge effects enhance carbon uptake and its vulnerability to climate change in temperate broadleaf forests. Proceedings of the National Academy of Sciences.

[CR57] Remm L, Lõhmus A, Leibak E, Kohv M, Salm JO, Lõhmus P, Rosenvald R, Runnel K (2019). Restoration dilemmas between future ecosystem and current species values: The concept and a practical approach in Estonian mires. Journal of environmental management.

[CR58] Root-Bernstein M, Ladle R (2019). Ecology of a widespread large omnivore, Homo sapiens, and its impacts on ecosystem processes. Ecology and evolution.

[CR59] Salmón E (2000). Kincentric ecology: indigenous perceptions of the human–nature relationship. Ecological Applications.

[CR60] Schepers F, Jepson P (2016). Rewilding in a European context. International Journal of Wilderness.

[CR61] Schläppy ML, Hobbs RJ (2019). A triage framework for managing novel, hybrid, and designed marine ecosystems. Global Change Biology.

[CR62] Scotney, R. 2014. Wilderness recognized: environments free from human control. In *Old World and New World Perspectives in Environmental Philosophy*, 73–90. Springer, Cham.

[CR63] Scottish Natural Heritage. 2014. Mapping of Scotland’s Wildness and Wild Land: Non–technical Description of the Methodology. https://www.nature.scot/snhs-mapping-scotlands-wildness-and-wild-land-non-technical-descirption-methodology. Accessed 23 June 2020

[CR64] Soulé M, Noss R (1998). Rewilding and biodiversity: complementary goals for continental conservation. Wild Earth.

[CR65] Suraci JP, Clinchy M, Zanette LY, Wilmers CC (2019). Fear of humans as apex predators has landscape-scale impacts from mountain lions to mice. Ecology Letters.

[CR66] Sweeney OF, Turnbull J, Jones M, Letnic M, Newsome TM, Sharp A (2019). An Australian perspective on rewilding. Conservation Biology.

[CR67] Thompson PL, Isbell F, Loreau M, O’connor MI, Gonzalez A (2018). The strength of the biodiversity–ecosystem function relationship depends on spatial scale. Proceedings of the Royal Society B.

[CR68] Torres A, Fernández N, Zu Ermgassen S, Helmer W, Revilla E, Saavedra D, Perino A, Mimet A (2018). Measuring rewilding progress. Philosophical Transactions of the Royal Society Biological Sciences.

[CR69] US Wilderness Act. 1964. Public Law 88–577, 16 U.S.C., 88th Congress, Second Session, September 3, 1964, 1131–1136.

[CR70] Van Meerbeek K, Muys B, Schowanek SD, Svenning JC (2019). Reconciling conflicting paradigms of biodiversity conservation: Human intervention and rewilding. BioScience.

[CR71] van den Bosch M, Sang ÅO (2017). Urban natural environments as nature-based solutions for improved public health–A systematic review of reviews. Environmental research.

[CR72] Vasile M (2018). The vulnerable bison: Practices and meanings of rewilding in the Romanian Carpathians. Conservation and Society.

[CR73] Wallace KJ, Wagner C, Smith MJ (2016). Eliciting human values for conservation planning and decisions: A global issue. Journal of Environmental Management.

[CR74] Ward K, Pettorelli N, Durant SM, Du Toit JT (2019). For wilderness or wildness? Decolonising rewilding. Rewilding.

[CR75] WCS. 2020. Rocky mountains. https://www.wcs.org/our-work/regions/rocky-mountains. Accessed 23 June 2020

[CR76] Wentworth J, Alison J (2016). Rewilding and Ecosystem Services.

[CR77] Wildlands Network. 2020. Frequently Asked Questions. https://wildlandsnetwork.org/frequently-asked-questions/. Accessed 23 June 2020

[CR78] Woodland Trust. 2017. Rewilding – the Woodland Trust’s position. https://www.woodlandtrust.org.uk/publications/2017/07/rewilding-position-statement/. Accessed 23 June 2020

