# Supplementary material for: The policy consequences of defining rewilding
Source: Ambio. 2021 May 13;51(1):93–102. doi: 10.1007/s13280-021-01560-8 (PMC8651963; doi:10.1007/s13280-021-01560-8)
Supplement: Supplementary file 1 — Supplementary material 1 (PDF 676 kb) [file 13280_2021_1560_MOESM1_ESM.pdf]

Ambio

Electronic Supplementary Material
This supplementary material has not been peer reviewed.

Title: **The policy consequences of defining rewilding**

Authors: Henrike Schulte to Bühne, Nathalie Pettorelli, Michael Hoffmann

Supplementary Materials

A) What are the different types of rewilding?

Rewilding proponents have developed a diverse range of rewilding types that are broadly distinguished by the type of intervention they propose in order to achieve rewilding goals (it is important to note that the goals they aim to achieve may also differ, see "What is wild?"). All approaches agree that the aim is to **minimise or remove active conservation interventions** in the long term; however, some approaches argue that particular active interventions may be necessary to kickstart the rewilding process. In the oldest definition of rewilding, Soulé & Noss (1998) highlight the need to protect large areas land, encompassing habitat cores and corridors, and restore populations of keystone species, emphasising the importance of large predators (also referred to as the 3 C's, or **cores, corridors, carnivores**). Retaining the focus on large, charismatic species, Donlan et al. (2006) proposed an approach termed **Pleistocene rewilding,** in which Pleistocene assemblages are used as a guide to what species should be reintroduced (in this case across North America). **Trophic rewilding** approaches emphasise the (re-)introduction of ecologically important species or their functional analogues (especially predators and large herbivores) to restore trophic networks and ecosystem function (Naundrup & Svenning 2015, Svenning et al. 2016). Though trophic rewilding does not refer to a particular era to define what "complete" trophic networks look like, it implicitly refers back to the period before large animals were extirpated (Jørgensen 2015). Definitions of **ecological rewilding**, i.e. the restoration of "natural" ecological and evolutionary processes to promote self-sustaining ecosystems (Stanley Price 2011, Lorimer et al. 2015, Perino et al. 2019), overlap with these definitions, but put more emphasis on restoring ecosystem functioning per se, rather than the means by which this is achieved. Thus, ecological rewilding could entail removing dams or firebreaks to allow "natural" disturbance regimes to develop, as well as species translocations, including plants (examples in Torres et al. 2018 and Pieck 2019). Like ecological rewilding, **passive rewilding** aims to create self-sustaining ecosystems, but emphasises the reduction of human control of the landscape, e.g. via land abandonment, as a fundamental means to achieve this (Höchtl. et al 2005, Pereira and Navarro 2015).

References

Donlan, J. C., Berger, J., Bock, C.E., Bock, J.H., Burney, D.A., Estes, J.A., Foreman, D., Martin, P.S., Roemer, G.W., Smith, F.A. and Soulé, M.E., 2006. Pleistocene rewilding: an optimistic agenda for twenty-first century conservation. *The American Naturalist*, *168*(5), pp.660-681.

Höchtl, F., Lehringer, S. and Konold, W., 2005. "Wilderness": what it means when it becomes a reality—a case study from the southwestern Alps. *Landscape and urban planning*, *70*(1-2), pp.85-95.

Jørgensen, D., 2015. Rethinking rewilding. *Geoforum*, *65*, pp.482-488.

Lorimer, J., Sandom, C., Jepson, P., Doughty, C., Barua, M. and Kirby, K.J., 2015. Rewilding: Science, practice, and politics. *Annual Review of Environment and Resources*, *40*, pp.39-62.

Naundrup, P.J. and Svenning, J.C., 2015. A geographic assessment of the global scope for rewilding with wild-living horses (Equus ferus). *PloS one*, *10*(7).

Pereira, H.M. and Navarro, L.M. eds., 2015. Preface. In: *Rewilding european landscapes*. Pereira, H.M. and Navarro, L.M. eds. New York: Springer International Publishing.

Perino, A., Pereira, H.M., Navarro, L.M., Fernández, N., Bullock, J.M., Ceaușu, S., Cortés-Avizanda, A., van Klink, R., Kuemmerle, T., Lomba, A. and Pe'er, G., 2019. Rewilding complex ecosystems. *Science*, *364*(6438), p.eaav5570.

Pieck, S.K., 2019. Conserving novel ecosystems and layered landscapes along the inter-German border. *Landscape Research*, pp.1-13.

Soulé, M. and Noss, R., 1998. Rewilding and biodiversity: complementary goals for continental conservation. *Wild Earth*, *8*, pp.18-28.

Stanley Price, M.R., 2011. Reintroductions in today's Arabian Peninsula: The first steps for a grander vision? *Zoology in the Middle East* (Supplement 3), pp.159-167

Svenning, J.C., Pedersen, P.B., Donlan, C.J., Ejrnæs, R., Faurby, S., Galetti, M., Hansen, D.M., Sandel, B., Sandom, C.J., Terborgh, J.W. and Vera, F.W., 2016. Science for a wilder Anthropocene: Synthesis and future directions for trophic rewilding research. *Proceedings of the National Academy of Sciences*, *113*(4), pp.898-906.

Torres, A., Fernández, N., Zu Ermgassen, S., Helmer, W., Revilla, E., Saavedra, D., Perino, A., Mimet, A., Rey-Benayas, J.M., Selva, N. and Schepers, F., 2018. Measuring rewilding progress. *Philosophical Transactions of the Royal Society B: Biological Sciences*, *373*(1761), p.20170433.
